# Consumption of Thermally Processed Meat Containing Carcinogenic Compounds (Polycyclic Aromatic Hydrocarbons and Heterocyclic Aromatic Amines) versus a Risk of Some Cancers in Humans and the Possibility of Reducing Their Formation by Natural Food Additives—A Literature Review

**DOI:** 10.3390/ijerph19084781

**Published:** 2022-04-14

**Authors:** Sylwia Bulanda, Beata Janoszka

**Affiliations:** Department of Chemistry, Faculty of Medical Sciences in Zabrze, Medical University of Silesia in Katowice, Jordana 19, 41-808 Zabrze, Poland

**Keywords:** cancer, meat, PAH, HAA, cooking procedures

## Abstract

(1) Background: Thermal treatment of high-protein food may lead to the formation of mutagenic and carcinogenic compounds, e.g., polycyclic aromatic hydrocarbons and heterocyclic aromatic amines. Frequent consumption of processed meat was classified by the International Agency for Research on Cancer as directly carcinogenic for humans. (2) Methods: A literature review was carried out based on a search of online databases for articles on consuming thermally processed meat containing carcinogenic compounds versus a risk of cancers in humans published between 2001 and 2021. (3) Results: A review of the current literature on the participation of PAHs and HAA in the formation of certain neoplasms indicates a positive relationship between diet and the incidences of many cancers, especially colon cancer. A simple way to obtain dishes with reduced contents of harmful compounds is the use of spices and vegetables as meat additives. These seasonings are usually rich in antioxidants that influence the mechanism of HAA and PAH synthesis in food. (4) Conclusions: As there is a growing risk of a cancer tendency because of exposing humans to PAHs and HAAs, it is extremely vital to find a simple way to limit carcinogenic compound synthesis in a processed proteinaceous food. Disseminating the knowledge about the conditions for preparing dishes with a reduced content of carcinogenic compounds could become a vital element of cancer prevention programs.

## 1. Introduction

It is assumed that 90–95% of cancers are caused by environmental factors. Even 30–35% of them are caused by a diet [1]. Food may contain many harmful chemical compounds left after the production process (nitrates, pesticides and dioxins). Compounds with mutagenic and carcinogenic activity may also be formed during storage or the thermal processing of high-protein food [2]. These compounds include products of lipid and protein oxidation, polycyclic aromatic hydrocarbons (PAHs) and heterocyclic aromatic amines (HAAs) and, also, nitroso compounds (NOCs) formed from the nitrates (III) added to meat products [3]. Carcinogenic NOCs may also be formed endogenously with the contribution of heme iron [3]. In 2015, the International Agency for Research on Cancer (IARC), based on a sufficient number of evidence for harmfulness for human health, classified red meat as “probably carcinogenic for humans” (group 2A) and processed red meat as “carcinogenic for humans” (group 1) [4]. To what extent the diet may cause a death from cancer depends on the type of cancer. Regular consumption of red and thermally treated meat is risky particularly in the case of the following cancers: colon, prostatic gland, breast, stomach, pancreas and oral cavity. In the present paper, a literature review on muta- and carcinogenic contributions of PAHs and HAAs contained in red meat in some cancer formations is presented.

## 2. Materials and Methods

### 2.1. Protocol Registration

**Figure 1:**
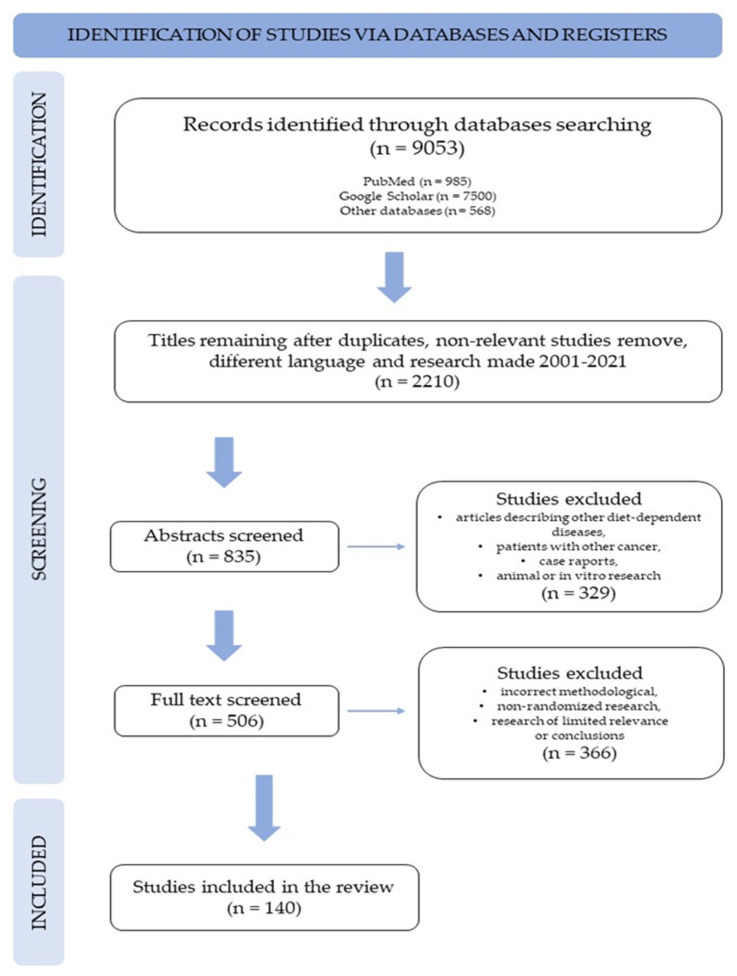
Protocol PRISMA flowcharts for the review.

In September 2021, the protocol for this review was developed. The review has been registered in the PROSPERO database (identification number 318707). For the methodological evaluation of the quality of this review, the authors used the AMSTER 2 checklist and the Preferred Reporting Items for Systematic Reviews and Meta-Analyzes (PRISMA) protocols (Figure 1).

### 2.2. Selection Criteria

The selection criteria were as follows: (1) case–control or cohort studies involving patients with specific cancers; (2) research works; (3) systematic reviews and meta-analyses; (4) research from the period 2001–2021; (5) research in English; (6) only studies involving humans were included in the analysis; (7) research about meat consumption that did not specifically contain red or processed meat were excluded from the analysis; and (8) excluded were also the articles describing other diet-dependent diseases. After applying the inclusion and exclusion criteria and analysis of all the abstracts, 140 articles were selected on the basis of which this work was written.

### 2.3. Search Strategy

A review of the literature was performed using the PubMed, Medline, Google Scholar, ProQuest, CINAHL and OpenGrey online databases. The following keywords were used to find the relevant articles: “red meat”, “polycyclic aromatic hydrocarbons”, “PAHs”, “heterocyclic aromatic amines”, ”HAA”, “HCA”, “cooking procedures”, “head and neck cancer”, “esophagus cancer”, “oesophagus cancer”, “pancreatic cancer”, “stomach cancer”, “colon cancer”, “prostate cancer”, “lymphoma”, “kidney cancer”, “bladder cancer”, “breast cancer”, “risk”, “prevention” and “diet”. The review concentrated on the publications from the period 2010–2021. In order to identify additional relevant literature, the databases were also searched manually. The sets of keywords were combined individually, and the eligibility of each study was judged independently by two authors.

## 3. Results

### 3.1. Polycyclic Aromatic Hydrocarbons (PAHs) in Food

Polycyclic aromatic hydrocarbons are a numerous group of over 200 organic compounds built of two or more fused aromatic rings [5]. PAHs are formed the during incomplete combustion of organic matter [6]. PAHs are slightly water-soluble. Due to a lipophilic character, they tend to gather in an alimentary chain [7].

Polycyclic aromatic hydrocarbons may get into food from the polluted environment and from the atmosphere adsorbed into particulate matter, as well as from water and soil. These compounds have been found in fruit and vegetables coming from the fields in industrial regions and the fields placed close to busy roads [8,9]. PAHs may be also formed during food processing. Protein products (fish and meat), which are smoked and dried or thermally cooked (grilled, roasted or fried), are a main source of polycyclic aromatic hydrocarbons [5,10,11,12,13,14,15]. Particles of organic components in food are easily fragmented under high temperatures during pyrolysis, and free radicals that are formed may create PAHs via pyrosynthesis [6,16]. Model testing showed that polycyclic aromatic hydrocarbons may be formed, among others, of aliphatic α-amino acids [17,18,19]. It was found that, in pork sausages containing more basic amino acids (L-lysine and L-arginine), more PAHs were formed than in the presence of acidic amino acids (L-glutamic and L-aspartate). Moreover, the addition of an aromatic amino acid generated fewer PAHs in the grilled product than by using other amino acids [20]. The addition of D-glucose can increase the PAH formation when compared with that of keto-based sugar (D-fructose) [18,20]. PAHs are also formed from fatty acids and fats [16,21]. The addition of different methyl esters of fatty acids (as lipid precursors) in heated meat model systems led significantly to an increase the PAH concentration [22,23]. PAHs may also be formed from the result of cholesterol and vegetable sterol pyrolysis [24].

In the European Union countries, there have been regulations for many years, and they define permissible concentrations of PAHs in some groceries. These regulations are updated all the time according to the results of numerous studies concerning the PAH determination in food. In 2006, one of the hydrocarbons—benzo(a)pyrene (BaP)—was pointed by the Scientific Committee on Food of the EU (SFC) as a marker for PAH presence and its carcinogenic activity in food [25]. The report of the European Food Safety Authority (EFSA) presenting PAH concentrations in about 10,000 different food samples showed that, in about 33% of the samples analyzed for the 15 SFC priority PAHs, other carcinogenic and genotoxic PAHs were detected, despite testing negative for BaP (concentration of BaP was below the limit of detection) [24]. Based on these data, the Scientific Panel on Contaminants in the Food Chain of EFSA concluded that benzo(a)pyrene is not a suitable marker for the occurrence of polycyclic aromatic hydrocarbons in food and that a system of four compounds (PAH4): benzo(a)pyrene (BaP), benz(a)anthracene (BaA), benzo(b)fluoranthene (BbFl) and chrysene (Chr) or eight compounds (PAH8): PAH4 + benzo(k)fluoranthene (BkF), benzo(ghi)perylene (BghiP), dibenzo(a,h)anthracene (DBahA) and indeno(1,2,3-cd)pyrene (IP) would be the most suitable indicators of PAHs in food. The formulas of these PAHs are presented in Table 1. The EFSA also concluded that a system of eight hydrocarbons (PAH8) would not provide much added value compared to a system of four compounds (PAH4) [26]. According to the regulations the concentration of BaP in smoked meat and smoked meat products should not exceed 5 µg/kg and PAH4 30 µg/kg, and since 1 September 2014, it should be reduced even to 2 µg and 12 µg/kg. Additionally, the regulations say that heat-treated meat and meat products (grilled and barbecued) sold to the final consumer cannot contain more than 5 µg/kg and PAH4 30 µg/kg. In many EU countries, in traditionally smoked meat and smoked meat products, the lower PAH levels (2 and 12 µg/kg) were not achievable by changing smoking practices, and that is why, since 2020, in some countries of the EU, higher acceptable levels: 5 µg/kg (for BaP) and 30 µg/kg (for PAH4) have been accepted again for local products [27].

### 3.2. Heterocyclic Aromatic Amines (HAAs) in Food

Heterocyclic aromatic amines are organic nitrogen compounds built from two or three condensed rings from which one is aromatic and the others are heterocyclic. All HAAs have one exocyclic amine group (-NH_2_), except for three compounds from this group [28,29,30]. Investigations on heterocyclic aromatic amines formed in high-protein food started in Japan in the 1970s of the 20th century [31,32]. Since then, the structures of over 30 amines have been determined [27,28,32,33,34,35].

HAAs are classified into two groups, depending on their formation temperature: polar (called also “thermic”), which are formed in temperatures from 100 °C to 250 °C (temperatures typical for the cooking, baking, frying and grilling of proteinaceous food), and non-polar, which are formed in higher temperatures. Polar compounds are formed in accordance with the Maillard reaction, from α-amino acids, reducing sugars (glucose, fructose and ribose) and creatine, all of them occurring naturally in meat [28,36]. This reaction occurs between the amine group of amino acids and the carbonyl group of sugars. The Maillard reaction involves both free radicals (pyridine and pyrazine radicals) and reactive carbonyl structures, so both the free radical pathway and a carbonyl pathway were proposed as mechanisms of HAA synthesis [29]. Non-polar HAAs (called also “pyrolytic”) are formed mainly from the result of the thermal decomposition of tryptophan and glutamic acid. The reactive fragments formed at high temperatures through radical reactions may condense to generate heterocyclic structures [35].

The concentrations of HAAs in different meat dishes range from 0 up to several dozen ng in 1 g of the product [28]. So far, no regulations have been introduced regarding the permissible content of HAAs in food. The kind and concentration of heterocyclic aromatic amines formed in food depend on many factors—first of all, on temperature, time of heating and kind of thermal processing [16,30,37,38]. Moreover, they also depend on the kind of meat, content of amino acids [39] and sugars [28] in it and on the kind of fats used for dish preparing [40] and the additives as well [28,30,36,41]. Heterocyclic aromatic amines are formed as early as after a few minutes of frying, and they occur even in slightly fried dishes. Small amounts of heterocyclic amines can also be found in cooked and stewed dishes [28,36].

The International Agency for Research on Cancer included one of HAA in the group 2A (probably carcinogenic to humans) and nine to the 2B group (possibly carcinogenic to humans). The names and formulas of these compounds are presented in Table 2.

### 3.3. Mechanisms of Bioactivation

#### 3.3.1. PAHs

**Figure 2:**
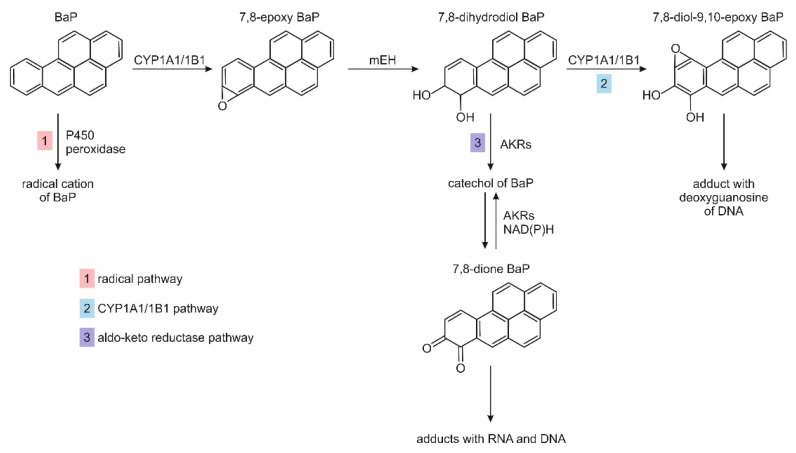
PAH metabolism pathway BaP based on references [44,45]. Abbreviations: CYP—cytochrome P450; mEH—microsomal epoxide hydrolase; AKRs—aldo-keto reductases; NAD(P)H—quinone oxidoreductase.

PAHs may enter the human body through the respiratory tract, digestive tract or the skin. As lipophilic compounds, they are transported in the blood connected with lipoproteins. To be removed from the body, they must undergo biotransformation to water-soluble compounds. The metabolism of PAHs proceeds in one or two phases with the participation of various liver enzymes. The reactions of phase I are catalyzed by cytochrome P450 (CYP) and leads to the formation of polar oxygen derivatives, amongst which epoxides are the primary products. The enzymatic processes of phase I include reactions of oxidations, reductions, hydrolyses and hydration. The phase II enzymes catalyze the conjugation of oxidized PAHs with compounds endogenously occurring in the body, such as sulphates, amino acids, glucuronic acid and glutathione (GSH). The conjugates (PAHs metabolites) as polar compounds may be excreted from the organism with bile and urine [43]. Compounds of carcinogenic activity can be generated from PAHs as a result of enzymatic reactions following three main pathways. The first occurs with the participation of cytochrome CYP1A1/1B1 and epoxide hydrolase, the second, undergone by CYP-peroxidase, is a radical pathway, and the third is catalyzed by aldo-keto reductases [44]. These three pathways for carcinogenic BaP are shown in Figure 2.

The products of PAH metabolic activation, i.e., radical cations, diol-epoxides and o-quinones, may form adducts with DNA. This can lead to errors in DNA replication and disturbance of the promoter methylation process or promoter binding. DNA mutation or abnormal gene expression can, finally, lead to tumor formation [44].

PAHs metabolites may also form adducts with proteins in cells, which may influence their normal activity. In addition, reactive oxygen species generated by PAH metabolites can initiate carcinogenesis by modifying DNA, lipids and proteins [44].

#### 3.3.2. HAAs

**Figure 3:**
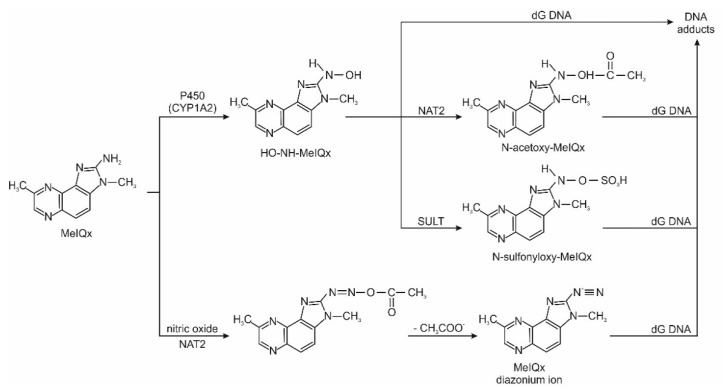
HAA metabolism pathway of MeIQx based on references [46,47]. Abbreviations: NAT2—N-acetyltransferases; SULT—sulfotransferases; dG—deoxyguanosine.

Heterocyclic aromatic amines are metabolically activated by catalytic cytochrome P450. In the first phase of activation, the exocyclic amino group of this compound is N hydroxylated into hydroxylamine (-NHOH). Then, with the participation of enzymes from the group of N-acetyltransferases and sulfotransferases, highly active metabolites (esters and sulphates) are formed, which combine with deoxyguanosine (dG) of DNA molecules or with protein molecules to form stable adducts. Adducts can cause errors in DNA replication, which can lead to mutations and, subsequently, to cancer development. Figure 3 shows the scheme of metabolic activation of the polar heterocyclic amine MeIQx. Simultaneously with microsomal activation, metabolic detoxification takes place in the liver. It consists of the oxidation to hydroxyl derivatives with the participation of cytochrome P450 isoenzymes. Next, as result of the enzyme-catalyzed reaction, sulphate and glucuronide esters are formed. Direct N-glucuronidation of the exocyclic amino group of HAAs is also possible. Polar metabolites resulting from detoxification can be excreted from the body via the urinary system.

### 3.4. Risk of Cancer

A high consumption of meat products is associated with an increased risk of cancer. The increased risk may be due to the high content of fat, protein or the formation of carcinogenic compounds, including heterocyclic aromatic amines and polycyclic aromatic hydrocarbons when processing meat at high temperatures [48]. In addition, heme iron, which is found in large quantities in red meat, may be involved in the endogenous formation of N-nitroso compounds from nitrates (III) [3,48]. These compounds can modify DNA synthesis, increase cell proliferation, affect hormone metabolism, increase insulin-like growth factors and contribute to the formation of free radicals, which leads to the development of cancer. [49].

#### 3.4.1. Head and Neck Cancer

Head and neck cancers (HNC) include, among others, oral cavity (OCC), oropharyngeal (OHPC) and laryngeal (LC) cancers. About 90% are squamous cell carcinomas. While the effects of tobacco, alcohol or papillomavirus on the increased risk of head and neck cancer are well-understood, the role of diet in HNC etiology is less clear. In a Dutch cohort study, the consumption of processed meat was positively associated with overall HNC and the HNC subtype OCC but not with OHPC and LC [50]. The study by Xu et al. found that eating processed meat contributed to the formation of mouth and oropharyngeal cancer, but no association was found between red meat and oropharyngeal cancer [51]. Li et al. confirmed data suggesting that the high consumption of red or processed meat is associated with an increased risk of nasopharyngeal carcinoma (NPC) [52].

#### 3.4.2. Gastrointestinal Cancer

Environmental factors, especially diet, play the most important role in the development of gastrointestinal neoplasms. Diet is estimated to contribute to 80% of colorectal cancer cases [53]. Liver is the major organ in the metabolism of PAHs. However, extrahepatic organs such as the gastrointestinal tract, spleen, lungs, heart, etc. may play a greater role. The underlying cause of gastrointestinal cancers is the information that human esophageal, duodenal and colon cells can metabolize PAHs and that microsomes from human gastric mucosa metabolize BaP [53]. Enzymes such as CYP1A1, CYP1A2, CYP1B1, glutathione S-transferase, UDP-glucuronosyl transferase and quinone oxidoreductase convert toxins into reactive metabolites that interact with cellular macromolecules, contributing to carcinogenesis [53].

##### Esophageal Cancer

Esophageal cancer (EC) is considered to be one of the most common types of cancer worldwide. It has been suggested that a high dietary protein intake increases the risk of EC [54]. Based on advanced case–control studies, Rostao et al. observed an increased risk of esophageal (squamous cell) cancer in people who have consumed large amounts of processed meat [55]. The above reports were confirmed by the meta-analysis by Choi et al., which showed an increase in the risk of esophageal cancer by 30% [56]. This association was stronger in esophageal adenocarcinoma than in squamous cell carcinoma [56]. Salehi et al. said that esophageal cancer is associated with the consumption of total red meat and processed meat but not with higher poultry consumption [57]. The study by Pournaghi et al. showed an association between the consumption of red meat, processed meat, poultry and the risk of EC. There was a positive relationship between the frequency of consuming red meat, processed meats (sausages), chicken with skin and the risk of EC; the use of lamb and fish showed no relationship [58]. In the study by Cross et al., high concentrations of two HAAs, MeIQx and PhIP, in meat consumed were associated with an increased risk of esophageal adenocarcinoma [59]. For esophageal neoplasms, the dose–response relationship between PAH concentration and EC risk is also important, suggesting a causal role of PAH exposure in the pathogenesis of EC [60].

##### Pancreatic Cancer

The relationship between the consumption of red and processed meat and the risk of pancreatic cancer is inconclusive. Malfatti et al. reported that mutagenic HAAs modify DNA molecules in pancreatic cells, indicating that heat-treated meat may be a risk factor for pancreatic cancer [61]. The study by Zhao et al. presented a case–control study that associated the consumption of red and processed meat with an increased risk of pancreatic cancer. Such a relationship was observed more often in men than in women [62]. Beaney et al. showed that the risk of pancreatic cancer, depending on the amount of meat consumed and the methods of its preparation, increases with age and concerns mainly people aged over 60 years [63].

##### Gastric Cancer

Gastric cancer is another cancer whose development is influenced by the PAHs and HAAs contained in food. The study by Cross et al. found a positive relationship between the consumption of 2-amino-3,4,8-trimethylimidazo[4,5-f]quinoxaline (DiMeIQx) and gastric carcinoma. Those with the highest consumption of DiMeIQx had an increased risk of gastric cardiac cancer, and benzo(a)pyrene showed no association [59]. The increased risk depends on the consumption of smoked, grilled and processed meat [64]. Additionally, the Western style of nutrition, which is dominated by a large amount of meat, increased the risk of gastric adenocarcinoma [65]. The association of an increased risk of gastric adenocarcinoma with high meat consumption was not demonstrated only in a study of the Netherlands population [66]. In addition, a meta-analysis by Zhao et al. suggested that there was no association between the consumption of red and processed meat and the risk of stomach cancer in cohort studies, although case–control studies have shown a positive correlation [67]. In conclusion, further well-designed prospective studies are needed to confirm the results of the influence of meat carcinogens on gastric cancer formation.

##### Colorectal Cancer

The incidence of colorectal cancer (CRC) depends on lifestyle, environmental factors, genetics and diet [2]. Exposure to food-derived HAAs and PAHs is considered to be an important factor in the development of CRC. Studies by Helmus et al. have confirmed that HAAs (mainly MeIQx and DiMeIQx) and PAHs found in red meat dishes are important factors initiating colon cancer carcinogenesis [68]. In the study by Cross et al., both red and processed meat consumption correlated positively with the development of colorectal cancer. These dependencies may result not only from exposure to HAAs (MeIQx and DiMeIQx), which is raised in meat prepared at high temperatures, but also from exposure to heme iron and nitrates contained in meat [69]. Steck et al. found positive links between the consumption of thermally processed meat and its HAAs compounds and colon cancer [70]. In addition, they investigated how a specific genotype modifies the association between meat consumption and meat-derived carcinogens and colon cancer [70]. Different genotypes show different expressions of the arising HAA–DNA adducts. The occurrence of the so-called “at-risk” alleles, which are predisposed toward the occurrence of CRC, is associated with a high consumption of red meat, well-done red meat, pan-fried red meat and meat carcinogens MeIQx and DiMeIQx [70].

The risk of diet-related cancer is also gender-modified. In the study by Vulcan et al., a tendency towards an increased risk of CRC with a higher total consumption of processed meat among men was observed. High beef consumption was associated with an increased risk of rectal cancer in men. Additionally, high pork consumption was associated with an increased risk of colon cancer in women [71]. In another work, a high beef and lamb consumption was associated with colon cancer risk but not with rectal cancer [72].

The authors of this study found one case–control study that did not support the hypothesis that the risk of colorectal cancer increases with the increasing consumption of red meat [73]. Multiple results, depending on the type of meat, sex and tumor location, show the complexity of colorectal cancer.

#### 3.4.3. Prostate Cancer

There is growing evidence that eating habits influence the incidence of prostate cancer. Heterocyclic amines are positively associated with the risk of prostate cancer in animal models [74]. However, the results are inconsistent in epidemiological studies. In the study by Sander et al., there was no association found between HAA consumption and advanced prostate cancer or between the high consumption of well-done meat and prostate cancer. Men showing the highest intake of PhIP, MeIQx and DiMeIQx did not have an increased risk of prostate cancer compared to men with the lowest intake [75]. On the other hand, in the study by John et al., an increased risk of prostate cancer was associated with the frequent consumption of grilled or well-done red meat. There was no increased risk of advanced prostate cancer with animal fat consumption, suggesting that the association between diets full of red meat and prostate cancer is not related to the fat content. It has been shown that the cooking method and the degree of roasting of the meat, affecting the concentration of carcinogenic HAAs and PAHs, determine the risk of prostate cancer [48]. Changes occur in the prostate gland that can induce DNA damage. Nakai et al. found that the all lobes of prostates are target tissues of PhIP-induced mutations. PhIP contributes to carcinogenesis by causing mutation and inflammation by acting as both an initiator and promoter of the tumor [76]. In the study by Bogen et al., the consumption of grilled red meat was associated with higher levels of PhIP–DNA adducts in prostate tumor cells in men who had undergone radical prostatectomy, and the consumption of PhIP from cooked meat determined increased prostatic-specific antigen (PSA) levels [77].

A meta-analysis by Fabiani et al. and a case–control study conducted in Argentina in 2008–2013 confirmed that eating patterns called “Western” and “carbohydrate”, characterized by high loads of red meat, processed meat, eggs, sweets, bread, pasta and rice, were significantly associated with an increased risk of PC [78,79]. It should be noted that the occurrence of PC is related not only to diet and lifestyle but also to family history.

#### 3.4.4. Lymphatic Cancer

There are many reports of an increased risk of lymphatic cancers related to a high consumption of processed high-protein products. Diet can influence the development of lymphoma by antigenic stimulation of the lymphoid tissue in the digestive tract by the actions of specific nutrients, resulting in changes in the immune system’s response [80].

Despite reports of increasing risk [81], the results regarding the association of a high consumption of red meat are not convincing about its involvement in non-Hodgkin’s lymphoma (NHL) risk, irrespective of the cooking method and degree of deep-frying that led to the formation of HAA and PAH [82]. The exception is a significant upward trend depending on the frequency of consumption of grilled or roasted chicken, the daily consumption of which was associated with an 80% increase in the risk of NHL [82]. Studies by Rohrmann et al. showed a positive association of chronic lymphocytic leukemia (CLL) with a high consumption of processed meat [83]. This was not confirmed by the study by Solans et al. [84].

#### 3.4.5. Renal and Bladder Cancer

Renal cell carcinoma (RCC), the most common kidney cancer in adults, originates in the lining of the proximal ileal tubule, where glucose, amino acids, uric acid and inorganic salts are reabsorbed into the filtrate [85]. Despite the role of the kidneys in the metabolism and urinary excretion of various compounds, studies of meat-derived mutagens and kidney cancer in humans are rare and limited by a small number of cases [86]. Daniel et al. examined the consumption of meat and meat-related mutagens in relation to RCC in a case–control study of US men and women. Exposure to PAH found in grilled meat was associated with a higher risk of kidney cancer. The risk of RCC increased with the consumption of grilled meat and PAHs, including benzo(a)pyrene. The risk of RCC was more than twice as high with the increasing BaP consumption [85].

The frequent consumption of processed meat increases the risk of bladder cancer, as shown in the National Institutes of Health (NIH) and the American Association of Retired Persons (AARP) Diet and Health Study. The results suggest a positive association between the frequent consumption of red meat and as dietary exposure to PhIP and bladder carcinogenicity [87].

#### 3.4.6. Breast Cancer

Dietary factors may increase the risk of breast cancer by modifying the levels of estrogens and other hormones (e.g., insulin-like growth factor). There is a hypothesis that the fats, iron and products of food processing (including HAAs) contained in meat modify the risk of breast cancer [88]. In the study by Genkinger et al., the statistically significant association between breast cancer and the consumption of red meat, processed meat, white meat or fish was not observed [88]. However, other research suggests that different types of breast cancers may have different etiologies. The frequent consumption of fried meat may increase the risk of, for example, ER +/PR- breast cancer [89]. Additionally, in Nurses’ Health Study II, a higher consumption of red meat was associated with almost twice the risk of ER +/PR + breast cancer [90]. However, no association was observed with culinary practices, exposure to heterocyclic amines or heme iron from red meat consumption with a risk of breast cancer in the studies by Lo et al. [91], contrary to the reports of Anderson et al., who found that the consumption of processed meat may increase the overall breast cancer risk [92].

## 4. Reducing the Risk of Cancer by Supplementing the Diet with Plant Products

Diet is the major source of PAHs and HAAs for the nonsmoking population, contributing to over 90% of the total exposure to these chemical compounds [8,12]. According to the guidelines of the National Food Agency in Sweden, the consumption of red meat should not exceed 500 g per week [93]. Above this value, an increased cancer risk from red meat is observed [71].

Meat and meat products, despite their proven carcinogenic potential, have high nutritional value. In addition to the main ingredients (i.e., amino acids; proteins with high biological value and minerals such as iron, zinc, selenium, manganese, vitamin B12 and other B vitamins), meat is rich in bioactive ingredients such as taurine, L-carnitine, choline, alpha-lipoic acid, linoleic acid, glutathione, creatine, coenzyme Q10 and bioactive peptides [94]. A person using a normal diet is not able to completely avoid exposure to muta- and carcinogenic compounds formed during the thermal processing of food. Although the concentrations of heterocyclic aromatic amines and polycyclic aromatic hydrocarbons in meat dishes are low, all treatments and processes leading to the reduction of their formation in food deserve attention. In order not to give up the nutritional properties of meat, it is worth modifying the methods of its thermal processing and, also, composing the diet in such a way as to limit the exposure of a human body to carcinogenic and mutagenic substances present in meat products. Recently, several extensive literature reviews have been published, the authors of which have made an attempt to systematize the knowledge of the formation of PAHs and HAAs in food and the possibility of limiting human exposure to muta- and carcinogenic compounds [5,12,95,96,97].

Many cancers can be prevented by meeting the dietary guidelines of cancer societies and recommendations for cancer prevention by optimizing the consumption and combination of certain foods. Numerous scientific reports indicate the chemopreventive (anticancer) effects of a diet rich in vegetables and fruits [49,64,65,85,98,99,100]. Such diets are associated with a reduced risk of chronic disease, inflammation and mortality [99]. The protective effect of a diet based on plant-based products may be due to their anti-inflammatory and antioxidant effects [101].

Plant-based foods are a rich source of antioxidants, phytoestrogens and flavonoids, especially flavanones, which can help prevent cancer growth through anti-inflammatory action, scavenging free radicals or blocking the formation of carcinogens [49]. Other ingredients in these products, including fiber, folate, vitamin C, vitamin A and beta-carotene, may also have anticancer effects [49]. Research confirms that a greater consumption of fruits and vegetables is associated with a significant reduction in the risk of a variety of malignancies, including cancers of the esophagus, lung, stomach and colon [49]. It has been proven that the strict adherence to Mediterranean dietary patterns allows one to prevent, e.g., gastric adenocarcinoma [65].

Maximova et al. showed that a low consumption of vegetables and fruits, with the simultaneous frequent consumption of highly processed meat products, leads to an increase in the incidence of cancer and a shorter time for the development of this disease [98].

The results of studies by Mouss et al. confirmed that the nutritional profile based on fruit and vegetables was inversely correlated with the risk of hepatocellular carcinoma (HCC) [99], esophageal cancer (EC) [49] and renal cell carcinoma (RCC) [85]. There was no such clear correlation between the consumption of whole grains and fiber and red meat in terms of cancer development [98], although a study by Anderson et al. demonstrated that a high-fiber intake protects against colon cancer (CRC) in people genetically susceptible to IL10-related CRC [102].

In addition, the results of studies by Yang et al. indicate that the reduction of the risk of CRC positively correlates with a diet with an increased content of fiber [103]. The results from The World Cancer Research Fund International project have shown that a consumption of 90 g/day of whole grain is associated with a reduced risk of colon cancer, mainly due to the fiber content in whole grains [104]. The authors should discuss the results and how they can be interpreted from the perspective of previous studies and of the working hypotheses. The findings and their implications should be discussed in the broadest context possible. Future research directions may also be highlighted.

## 5. Influence of Vegetable Additives on the Synthesis of Carcinogenic PAHs and HAAs in Thermally Processed Meat Dishes

Vegetable additives not only improve the taste of meat dishes but also, as the latest scientific research shows, can decrease the contents of some xenobiotics. These additives, usually rich in antioxidants, can modify the free radical mechanisms of HAA and PAH synthesis in food [29,30,41,105,106].

The results of the investigations on the influence of the natural additives and antioxidants contained in them on PAH formation in meat dishes are presented in Table 3. Some studies tried not only to evaluate the possibility of the reduction of the compounds formation in meat dishes but to also know the processes that take place under the influence of antioxidants. To achieve this, the phenolic profiles, total antioxidant status or radical scavenging activity were determined [97,107,108,109].

An effective way to lower the PAH concentration in grilled dishes is to marinate the meat. Marinades containing lemon juice [110], as well as vitamins E and C [111], decreased the PAH levels by even 70% [110].

Marinating in beer significantly lowered the PAH contents in grilled meat, and the investigations showed that black beer had the highest inhibitory effect on the formation of PAH8 (53%) in charcoal-grilled pork, while pilsner alcoholic beer had the lowest (13%). The inhibitory effect of beer marinades on PAH increased with the increase of their radical scavenging activity [107,109] and the higher levels of the phenolic compounds [109].

A significant inhibitory effect of fruit/wine vinegars (sprayed on meat before grilling) on the formation of polycyclic aromatic hydrocarbons in charcoal-grilled pork was also shown. However, the mechanism of PAH reduction by vinegars is complex and probably depends on the phenolic profiles, pH and the interactions that can occur between meat and vinegar components [112].

The influence of green tea and yerba mate marinades on BaP formation in grilled and roasted meat was investigated [113,114]. Although benzo(a)pyrene was found in all samples, the tea marinades reduced the activity of the radicals and lipid oxidation [113,114].

It was proven that the addition of onion or garlic to pork meat being fried was able to decrease the PAH concentration [100]. Recent studies have shown that garlic and garlic essential oil added to charcoal-grilled pork sausages significantly decreased the BaP concentration [108], with inhibition being dependent on the number of sulfur (-S-) and thioallyl groups (–S–CH_2_–CH=CH_2_) in sulfide compounds. The mechanism of sulfides influencing BaP formation was related to the free radical reaction.

The addition of spices of high antioxidant capacity (i.e., paprika, ginger and black pepper) to thermally processed meat can decrease PAH and HAA contents, irrespective of the kind of meat [111]. It was also proven that the use of curcuma, lemon grass and curry leaves during meat roasting causes PAH and HAA concentrations to decrease [110,115,116].

Lately, a review of studies on the effects of cooking techniques and spiced marinades on the formation of PAHs, as well as heterocyclic amines in meats, has been published [97]. A meta-analysis of the results showed that the garlic and onion, pepper and other spices with phenolic compounds inhibited the formation of HAAs and PAHs due to the antioxidant and electron transfer mechanism.

The results of the studies on the effects of marinades, spices and additives often used for meat dish preparations on HAA synthesis are presented in Table 4. An effective way to lower the HAA concentrations in meat products is the use of natural additives containing flavonoids, vitamins C and E and catechin.

A model study showed that the inhibitory effect of flavonoids depended mainly on their hydrophobicity, the position of the hydroxyl groups and topological structure. Catechin was the most effective inhibitor of HAA, followed by luteolin and genistein [105]. Other studies have shown that resveratrol was found to be the most efficient, as it totally inhibited MeIQ and reduced MeIQx and PhIP formation by 40 and 70%, respectively [115]. Antioxidants can inhibit various Maillard reaction pathways. They prevent the formation of HAAs through free radical quenching and scavenging. It was found that phenolic compounds are able to scavenge a wide range of reactive carbonyls formed during proteinaceous food thermal treating, even under common cooking conditions [29,105].

It is worth saying that not only meat additives have an effect on the concentration of carcinogenic substances in food product but also the way of preparation, i.e., methods of smoking (direct or indirect), the heat source, the smoke generation process (pyrolysis and air flow temperature), the distance between the food and the heat source and the fat content in a product [117]. Some alternative technologies may be applied, such as high-pressure treatment, cold plasma and ultrasounds [118]. Moreover, to limit HAA formation in food, long high-temperature thermal processes for food preparation should be avoided and, also, the dripping of fat during meat grilling.

**Table 3:** Influence of marinating and spices or natural plant additives on PAH concentrations in cooked meat. Concentration changes are presented as given in the publications, i.e., in % or ng/g.

Additives	Sample Type and Heat Treatment Conditions	Influence on BaP Concentration	Influence on PAHs Concentration	Reference
**Meat model system**				
Epigallocatechin gallate (EGCG), (butylated hydroxyanisole (BHA), 3,5-di-tert-4-butylhydroxytoluene (BHT), α-tocopherol, sesamol (200 ng/g)	Meat model system heated at 200 °C for 30 min (dry conditions)	Control 6.6 ng/g. Maximum decrease: to 4.1 ng/g (sesamol)	PAH8 Control 22.3 ng/g. Decrease range: from 14.4 (EGCG) to 11.6 ng/g (sesamol)	[22]
**Marinades**				
Three phenolic acid marinades with: protocatechuic acid (PA), gallic acid (GA) and ferulic acid (FA) (0.1–5 mg/mL)	Charcoal-grilled chicken wings	Control 3.3 ng/g. Decrease range: 2.95 ng/g (0.1 mg/mL GA) to 2.1 ng/mL (3 mg/mL FA)	PAH8 Control 12.83 ng/g. Decrease range: 12.3 (0.1 mg/mL FA) to 7.7 (3 mg/mL PA)	[119]
Marinades with 8 phenolic compounds existing in green tea: (epigallocatechin gallate (EGCG), gallocatechin (GC), catechin (C), epicatechin gallate 107 (ECG), catechin gallate (CG), naringenin, and quinic acid (QA)	Charcoal-grilled chicken wings	Control 1.5 ng/g. Reduction in the range from 20.5% (GC) to 71% (QA)	PAH8 Control 2,5 ng/g. Reduction range from 15% (GC) to 54.5% (QA)	[120]
Black beer, alcoholic and non-alcoholic pilsner beer marinades,	Charcoal-grilled pork	Control 2.7 ng/g. Decrease range: 2.2 ng/g (alcoholic beer) to 1.1 ng/g (black beer)	PAH8 Control 20.7 ng/g. Decrease range: 17.8 ng/g (alcoholic beer) to 9.7 ng/g (black beer)	[107]
Six brands of beer marinades and eleven phenolic compounds (e.g., gallic acid, hydroxycinnamic acids (ferulic acid), and flavonoids (catechin), Homovanillic acid (HVA)	Charcoal-grilled chicken wings	Control 2.3 ng/g. Decrease to 0.5 ng/g (Heineken); Increase to 2.8 (Snow) All phenolic compounds decreased the BaP concentration. Maximum–HVA (67%)	PAH8 Control 13.0 ng/g. Decrease to 4.3 (Heineken) Increase to 18.1 ng/g (Snow) All phenolic compounds decreased the PAH8 concentration. Maximum–HVA (48%)	[109]
Tea marinade with green tea (GT) and yerba mate (YM) (1%)	Charcoal-grilled pork belly	Reduction: 24.5% (GT) and 31.5% (YM)	-	[113]
Meat sprayed with vinegars: - white wine vinegar (WWV), - red wine vinegar (RWV), - apple cider vinegar (ACV), - elderberry vinegar (EV), - apple cider vinegar with raspberry juice (ACVR)	Charcoal-grilled pork	Control 3.4 ng/g. Reduction in the range from 58.5 (ACVR) to 85.3% (WWV)	PAH4 Control 31.5 ng/g. Reduction in the range from 55 (ACVR) to 82% (EV)	[112]
**Spices and natural plant additives**				
Gochujang (Korean Red Pepper Paste)	Charcoal-Grilled Pork belly	Reduction by 32%	16 PAH Reduction by (63.1%)	[121]
Black pepper, garlic, ginger, onion, paprika (P), and red chilli (0.5%)	Fried beef and chicken meatballs	Reduction in the range from 44% (paprika in chicken) to 100% (ginger in beef)	2 PAHs: BaA and BaP Reduction in the range from 47% (black pepper, beef) to 98% (ginger, beef)	[111]
Garlic (0.05–0.15%, *w*/*w*); garlic essential oil (GEO) (0.002–0.006%, *w*/*w*)	Charcoal-grilled pork sausages	Reduction: 37.2–62.3% (garlic); 29.1–57.1% (GEO)	-	[108]
Garlic (15%); onion (30%)	Pan fried pork (collars, chops)	Reduction: 55–71% (garlic); 44–74.5% (onion)	6 PAHs (BaA, BaP, BbF, BghiP, BkF, DBahA) reduction 41–66% (garlic); 3.5–67% (onion)	[100]

**Table 4:** Influence of marinating and spices or natural plant additives on HAA concentrations in cooked meat. Concentration changes are presented as given in the publications, i.e., in % or ng/g.

Additives	Sample Type and Heat Treatment Conditions	Reduction of HAAs Concentration	No Reduction or Increase of HAA Concentration	Reference
**Marinades**				
Marinade with 1% green tea extract	Pan-fried beef	PhIP reduction from 33.8 to 8.8 and AαC-from 14.7 ng/g to 2.2	4,8-DiMeIQx and MeIQx	[122]
Green (GT), oolong (OT) and white tea (WT) extracts (1%)	Grilled chicken drumsticks	IQ, PhIP, AαC, Harman, norharman Reduction 23% (WT), 16–18% for GT and OT	-	[114]
Beer (B) or red wine (RW) marinades	Pan-fried beef	PhIP–88% (B and RW); MeIQx–44% (B) and 33% (RW) AαC-7–77% (B, RW)	-	[123]
Black beer (BB), alcoholic (AB) and nonalcoholic pilsner beer (NAB) marinades	Charcoal-grilled pork	PhIP reduction from 6.1 ng/g to 1.6 (BB) Trp-P-1 from 5.9 ng/g to 0 ng/g (all marinades); AαC from 1.5 to 0.3 ng/g (BB); 4,8-DiMeIQx from 4.6 ng/g to 0 (BB)	4,8-DiMeIQx, MeAαC (NAB)	[124]
Complex (purchased) marinades based on acerola, oregano and sumac, with many other ingredients	Barbecued pork chops	Reduction by using each marinade: MeIQx from 1.11 to 0.73 ng/g; 4,8-DiMeIQx from 1.54 ng/g to 0.13 and PhIP from 11.35 to 0.13 ng/g	Harman and norharman	[125]
**Spices and natural plant additives**				
Rosemary ethanolic extracts (0.05–0.5%)	Cooked beef patties	MeIQx (92%) and PhIP (85%)	-	[126]
Oregano (0.25% and 0.5%)	Pan-fried ground beef patties	MeIQ (reduction 100%), MeIQx from 7.2 ng/g to 4.6 ng/g; PhIP from 2.3 ng/g to 1 ng/g	-	[115]
Black cumin (1%)	Cooked meatballs (at 250 °C)	MeIQx reduction from 1.53 ng/g to 0.86 ng/g; PhIP from 2.75 ng/g to 1.50 ng/g	-	[127]
Basil (1%)	Cooked meatballs (at 250 °C)	MeIQx reduction from 0.63 ng/g to 0.53 ng/g, MeIQ from 0.09 to 0.07 ng/g, 4,8-MeIQx, and PhIP–100% reduction	-	[116]
Turmeric, curry leaf, torch ginger and lemon grass	Grilled beef	Total 9 HAAs (IQ, IQx, MeIQ, MeIQx, 7,8-DiMeIQx, PhIP, Harman, Norharman, AαC) reduction in the range from 21% (curry leaf) to 94.7% (turmeric and lemon grass) (50:50 *w*/*w*)	-	[128]
Turmeric powder (0.5%)	Chicken meatballs	Total HAAs reduction by 72%	-	[129]
Black pepper (0.5; 1%, 1.5%)	Fried tilapia fillets	PhIP and MeIQx (reduction 100% by using 1% pepper)	MeIQ and norharman	[130]
Sichuan pepper (0.5%; 1%)	Grilled ground beef patties	PhIP reduction (by using 0.5% pepper) 82%, IQx 61%, MeIQx 28% and 4,8-DiMeIQx 79%	Harman and norharman	[131]
Black pepper, garlic, ginger, onion, paprika (P), and red chilli (0.5%)	Fried beef and chicken meatballs	IQ, MeIQ,4,8-DiMeIQx, PhIP. Inhibitory efficiency of the 4 HAAs in the range from 43% (onion) to 87% (ginger).	-	[111]
Chilli pepper (0.5%)	Roast beef patties	PhIP reduction: 68%; Total 8HAAs reduction: 46%	MeIQx, harman	[132]
Garlic (15%); onion (30%)	Pan fried pork (collars, chops)	IQ, MeIQ, MeIQx, 4,8-DiMeIQx, PhIP Total reduction in the range from 21 to 49.5% (onion) and 26–36 (garlic)		[133]
Beetroot juice (3%)	Meat-protein model system	PhIP reduction 60%, MeIQx 77% and IQ 87%	-	[134]
Cherry tissue (11.5%)	Fried ground beef patties	PhIP reduction in the range from 87–93%	-	[135]
Dried apple peel extract (0.1, 0.15 and 0.3%)	Pan fried beef patties	MeIQx reduction 41- 68%; 4,8-DiMeIQx 21–56%; PhIP 60–83%	-	[136]
Pomegranate seed extract (0.5%)	Beef and chicken meatballs (oven roasted, pan cooked, charcoal-barbecued, deep-fat fried)	The highest reduction: PhIP 68–75%; norharman 24–57%; harman 18–28%; IQ 45–46%; MeIQx 49–57%	Norharman and harman in beef oven roasted; IQ and MeIQx in chicken oven roasted and pan cooked	[137]
Hawthorns extract (0.5, and 1%)	Beef and chicken breast oven and pan-cooked	Total amount of 12 HAAs (Q, IQx, MeIQ, MeIQx, 4,8-DiMeIQx, 7,8-DiMeIQx, PhIP, harman, norharman, AαC, MeAαC, and Trp-P-2) reduced in the range of 12–100%	Harman	[138]
Apple skin and olive extracts, onion powder (1 and 3%)	Ground beef patties-grilled	MeIQx 49–51% and PhIP 51–65% (olive and apple extracts); MeIQx and PhIP 47 and 80.7%, (onion powder)	MeIQx (by using 1% onion) and PhIP (1% apple skin extract)	[139]

## 6. Conclusions

As there is a growing tendency for the consumption of processed food accompanied by the aging of the population, which means a longer exposure of humans to PAHs and HAAs, it is extremely vital to find a simple way of how to limit carcinogenic compound synthesis in a processed proteinaceous food.

The investigations that were reviewed showed that simple cooking processes when some additives rich in phenolic compounds are added to the food are a natural and effective way for the inhibition of the harmful compound formations, including PAHs and HAAs, in thermally treated meat.

The studies confirmed that a higher intake of total meat, red or processed meats is associated with a higher risk of cancer. Nevertheless, cohort studies are necessary in order to have a clear perspective for the long-term effect of a regular intake of xenobiotics formed during food processing on cancer generation. The consumption of vegetables even as meat additives demonstrates a protective activity against cancer. A diet rich in natural products (fruits and vegetables) may have a positive influence on one’s health due to antioxidants and their detoxifying properties [140].

## Figures and Tables

**Table 1:** Compounds of polycyclic aromatic hydrocarbons selected by EFSA as markers for the occurrence of PAHs in food [26].

Name	Abbreviation	Structure	IARC Carcinogenic Group *	Classification	
Benzo(a)pyrene	BaP	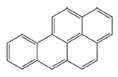	1	PAH4	PAH8
Benz(a)anthracene	BaA	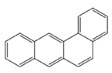	2B		
Benzo(b)fluoranthene	BbF	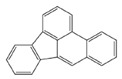	2B		
Chrysene	Chr	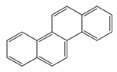	2B		
Benzo(k)fluoranthene	BkF	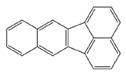	2B		
Benzo(ghi)perylene	BghiP	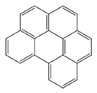	3		
Dibenzo(a,h)anthracene	DB(ah)A	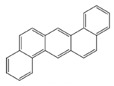	2A		
Indeno(1,2,3-cd)pyrene	IP	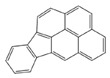	2B		

* IARC classification groups: 1—carcinogenic to humans; 2A—probably carcinogenic to humans; 2B—possibly carcinogenic to humans; 3—not classifiable as to its carcinogenicity to humans.

**Table 2:** Heterocyclic aromatic amines classified by IARC as carcinogenic [42].

Name	Abbreviation	Structure	IARC Carcinogenic Group *
**Polar HAAs (“thermic compounds”)**			
2-amino-3-methylimidazo [4,5-f]quinolone	IQ	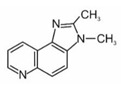	2A
2-amino-3,4-dimethylimidazo [4,5-f]quinoline	MeIQ	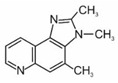	2B
2-amino-3,8-dimethylimidazo [4,5-f]quinoxaline	MeIQx	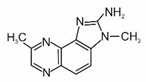	2B
2-amino-1-methyl-6-phenylimidazo [4,5-b]pyridine	PhIP	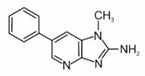	2B
**Non-polar HAAs (“pyrolytic compounds”)**			
2-amino-9H-pyrido [2,3-b]indole	AαC	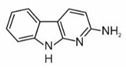	2B
2-amino-3-methyl-9H-pyrido [2,3-b]indole	MeAαC	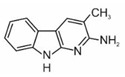	2B
3-amino-1-methyl-5H-pyrido [4,3-b]indole	Trp-P-2	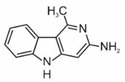	2B
3-amino-1,4-dimethyl-5H-pyrido (4,3-b)indole	Trp-P-1	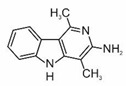	2B
2-aminodipyrido [1,2-a:3′,2′-d] imidazole	Glu-P-2	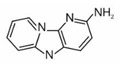	2B
2-amino-6-methyldipyrido [1,2-a:3′,2′ d] imidazole	Glu-P-1	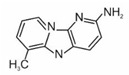	2B

* IARC classification groups: 2A—probably carcinogenic to humans, 2B—possibly carcinogenic to humans.
